# Morphological development of the dorsal hindbrain in an elasmobranch fish (*Leucoraja erinacea*)

**DOI:** 10.1186/s40851-018-0111-1

**Published:** 2018-11-10

**Authors:** Christos Michael Suriano, David Bodznick

**Affiliations:** 1 Biology Department, Wesleyan University, Middletown, CT 06459 USA; 2 Princeton Neuroscience Institute, Princeton University, Princeton, NJ 08540 USA

**Keywords:** Development, Elasmobranch, Cerebellum, Cerebellum-like structure, Morphology

## Abstract

The developmental anatomy of the dorsal hindbrain in an elasmobranch fish, *Leucoraja erinacea*, is described. We focus on the cerebellum, which is a synapomorphy for gnathostomes. Cerebellar development in *L. erinacea*, a representative of the most basal gnathostome lineage, may be a proxy for the ancestral state of cerebellar development. We also focus on sensory processing regions termed ‘cerebellum-like’ structures due to common anatomical and physiological features with the cerebellum. These structures may be considered generatively homologous if they share common developmental features. To test this hypothesis, the morphological development of the cerebellum and cerebellum-like structures must first be described. Of particular importance is the development of common features, such as the molecular layer, which is the defining characteristic of these structures. The molecular layers of the cerebellum and cerebellum-like structures are supplied with parallel fiber axons from distinct granule cell populations. These are the lateral granule mass, the dorsal granular ridge, the medial granule mass, and the granular eminences of the cerebellum. Cerebellar and cerebellar-like development in *L. erinacea* is similar to development in other elasmobranchs. The temporal order in which these granule cell populations develop suggests an evolutionary history of duplication or expansion of an existing developmental event.

## Research Highlights

The morphological development of the cerebellum and cerebellum-like structures in *Leucoraja erinacea* is described and further discussed in an evolutionary and comparative context to other elasmobranchs.

## Introduction

Here, we undertake a descriptive developmental analysis of the cerebellum and regions termed cerebellum-like structures in the dorsal rhombencephalon of the elasmobranch fish, *Leucoraja erinacea*. We focus on the development of the cerebellum because it is a synapomorphy for gnathostomes. Elasmobranchs are the most basal branch of this diverse clade and thus cerebellar development may be a proxy for the ancestral state of other jawed vertebrates. The cerebellum has important motor functions in addition to its less-defined roles in sensory processing and expectation of reward [1, 2].

We also focus on sensory processing nuclei present in several vertebrate lineages that are termed cerebellum-like structures due to shared anatomical, physiological and developmental features with the cerebellum. Elasmobranchs possess two cerebellum-like structures, termed the medial octavolateralis nucleus (MON) and the dorsal octavolateralis nucleus (DON). The MON and DON are the primary sensory nuclei of the mechanosensory lateral line and electrosensory systems, respectively [3]. These cerebellum-like structures may share a developmental program with the cerebellum and thus be considered generatively homologous.

The term generative homology describes a condition similar to serial homology, but emphasizes the developmental relationship between said structures [4]. The cerebellum and cerebellum-like structures develop from neighboring rhombomeres, so the genesis of these structures may occur through either a temporal or spatial expansion of otherwise restricted gene expression [5]. To better understand whether the cerebellum and cerebellum-like structures are generatively homologous, their development must be described both in terms of shared gene expression and morphological development. Features of the total shared cerebellar and cerebellar-like genetic toolkit have already been described in skates [6]. Although understanding shared gene expression profiles is important for resolving the question of generative homology of cerebellum and cerebellum-like structures, a comparative study of the morphological development must be completed in the skate. Characterization of common structures that are central to neuronal function is central to achieving a better understanding of shared aspects of cerebellar and cerebellar-like development.

Among the many shared anatomical, physiological and developmental characteristics, the defining feature of both the cerebellum and cerebellum-like structures is the presence of a distinctive molecular layer. This molecular layer is comprised of (1) parallel fiber axons that emanate from a granule cell population, (2) spiny apical dendrites of principal cells, and (3) inhibitory interneurons [7]. Principal cells, as defined here, are the major ascending projection cells of the nuclei and receive inputs from both parallel fibers and primary sensory afferents or climbing fibers. The molecular layer of the cerebellum, MON, and DON each receive parallel fiber input from a distinct granule cell population.

**Fig. 1:**
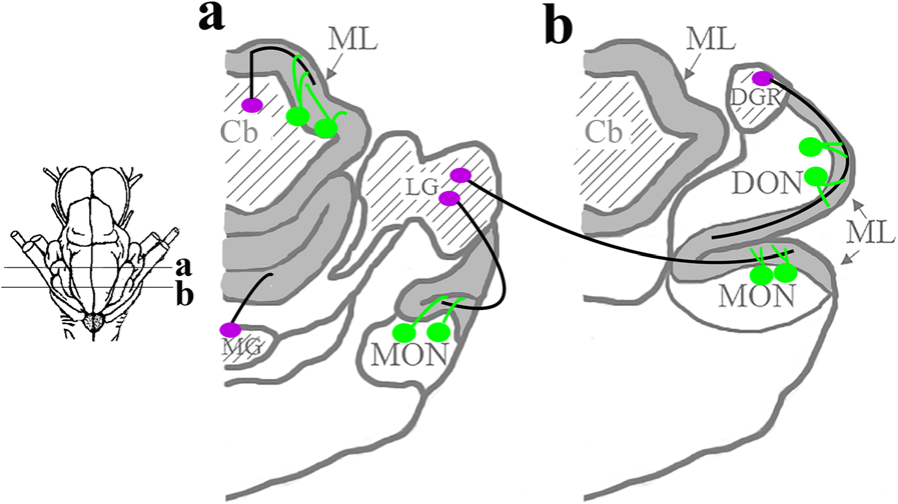
Anatomical schematic of *Leucoraja erinacea,* showing the projections of parallel fiber axons and their synapses with the spiny apical dendrites of principal cells in the molecular layers. Principal cells are green, granule cell somata are purple, parallel fiber axons are black, molecular layers are dark grey, and granule cell masses are marked by light grey. Abbreviations: *Cb*, cerebellum; *DGR*, dorsal granular ridge; *DON*, dorsal nucleus; *LG*, lateral granule cell mass; *MG*, medial granule cell mass; *MON*, medial nucleus

The lateral granule mass (LG) supplies parallel fibers to the MON, and the dorsal granular ridge (DGR) supplies the DON [8]. A distinct mass of granule cells, termed the granular eminences (GE) supply the molecular layer for most of the cerebellum except for the caudal most portion. This region, termed the vestibular cerebellum, receives parallel fibers from the medial granule cell mass (MG) (Fig. 1). The DGR and LG form a continuous population of granule cells that is also continuous with the more rostral GE and MG of the cerebellum.

It is possible that the GE and MG of the cerebellum and the LG and the DGR of the cerebellum-like structures, with their respective molecular layers, are generatively homologous and evolved by a duplication or expansion of a developmental event [9]. To better understand their evolutionary and developmental relationship, a clear description of cerebellar and cerebellar-like development is necessary. This is also necessary for a complete comparative analysis of cerebellar development across gnathostomes. While the anatomy of the cerebellum and cerebellum-like structures of adult elasmobranchs has been described in detail [8, 10] and some effort has been made to describe their development in sharks [5, 11], the development of these structures in skates has not yet been described. Here, we describe the morphological development of the cerebellar and cerebellar-like molecular layers and their respective granule cell masses, compare skate hindbrain development to other elasmobranchs and discuss the evolutionary implications of dorsal hindbrain development in the skate.

## Materials and methods

### Embryo collection

**Fig. 2:**
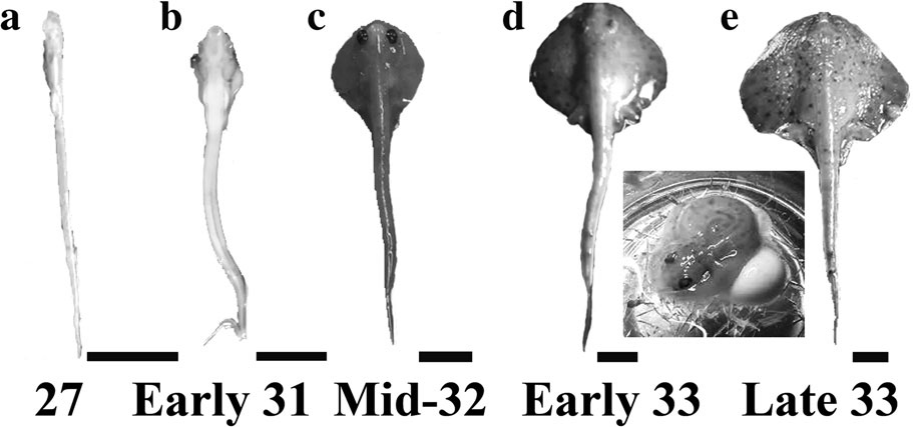
Whole mount images of *Leucoraja erinacea* at (**a**) stage 27, (**b**) early stage 31, (**c**) mid-stage 32, (**d**) early stage 33 and (**e**) late stage 33. Inset shows early stage 33 embryo with yolk. Scale bars equal 1 cm

Wild-caught adult little skates (*Leucoraja erinacea*) were obtained from the Marine Biological Laboratory in Woods Hole, MA. Skate eggs were collected weeks to months after adults were introduced to holding tanks at Wesleyan University. All holding tanks were held at 14 °C and salinity was maintained at a specific gravity of 1.021. Embryos were staged based on external embryonic morphology [12] and elasmobranch neural morphology [5], following the current literature. One specimen of the following stages was used for analysis: stage 27, early stage 31, mid-stage 32, early stage 33 and late stage 33 (Fig. 2). The research reported here was performed under guidelines established by the Wesleyan University IACUC.

### Cresyl violet acetate stain

Animals of varying embryological ages were staged and then anesthetized and euthanized by an overdose of benzocaine. The rostral half of animals was dissected and dehydrated through 15, 30, 50, 70, 95 and 100% ethyl alcohol. Specimens were then embedded in paraffin through a 3:1, 1:1 and 1:3 ethyl alcohol: xylene ratio and a 3:1, 1:1 and 1:3 xylene: paraffin ratio. Specimens were left in molten paraffin overnight and embedded the next day. The samples were sectioned at 10 μm by microtome and mounted on slides, which went through a rehydration series in 100% xylene, 100, 95, 70, 50% ethyl alcohol and PBS. Slides were then placed in cresyl violet acetate for two minutes and dehydrated through a quick progression of PBS to 50, 70, 95, 100% ethyl alcohol and cleared in xylene for 10 min. Slides were coverslipped and images were taken on a bright field microscope. Images were assembled in Adobe Photoshop for analysis.

## Results

### Stage 27

In *Leucoraja*, at embryonic stage 27 the eyes are visible, but present laterally and are unpigmented. The lateral line is visible around the eye. The mouth is open and heart shaped. The gill filaments are slightly protruding, but short and unvascularized. The pectoral fin lobes are visible, while the pelvic fins and dorsal fins are not as developed (Fig. 2a) [12].

**Fig. 3:**
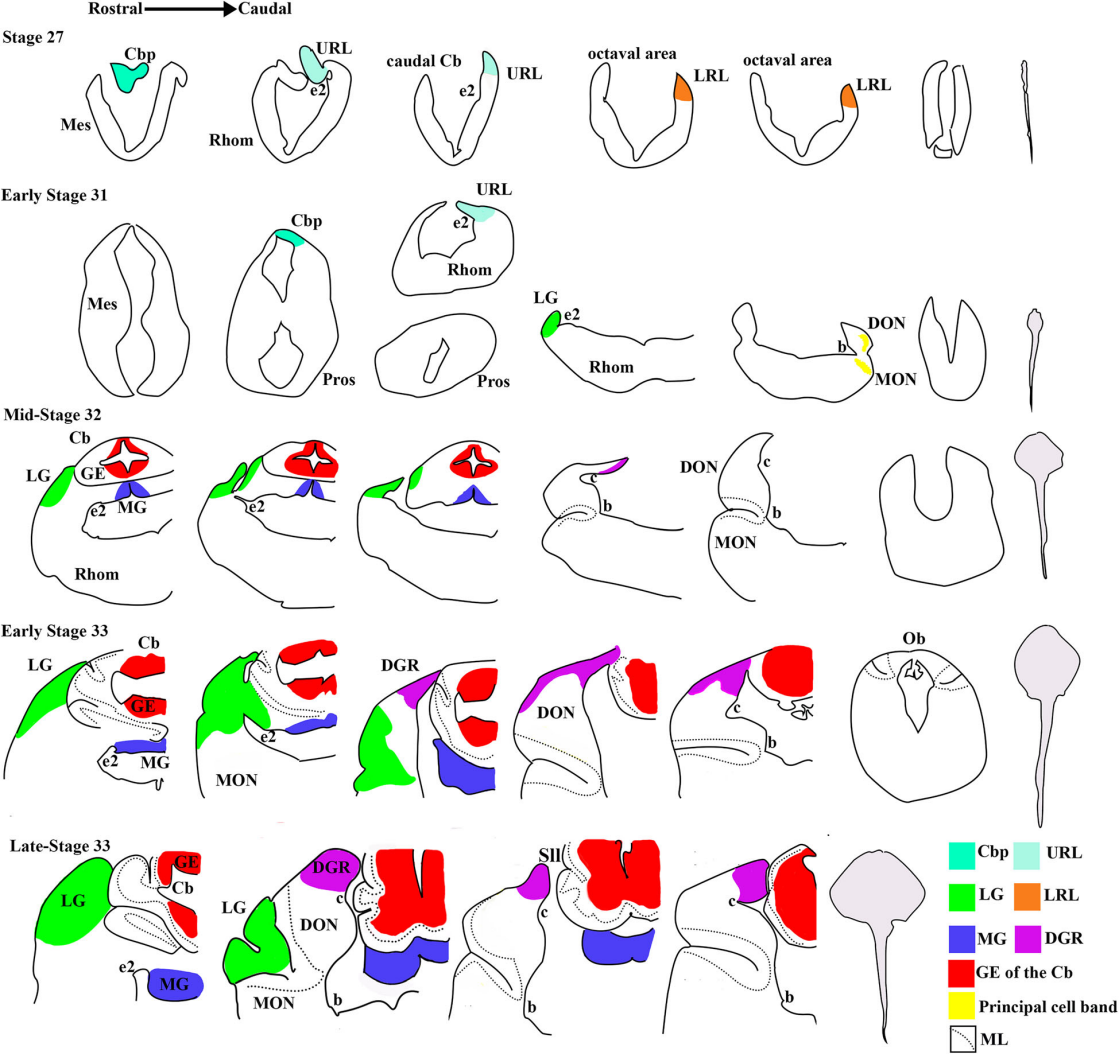
*Leucoraja erinacea,* anatomical drawing of the brain of at several developmental stages. Representative sections are described in a rostral to caudal manner. Brain regions are color coded (see legend). Outlines of whole specimen morphology is drawn to scale in grey. Brain regions are not drawn to scale. Abbreviations: b, sulcus b; c, sulcus c; Cb, cerebellum; *Cbp*, cerebellar plate; *DGR*, dorsal granular ridge; *DON*, dorsal nucleus; e2, sulcus e2; *GE*, granular eminences; *LG*, lateral granule mass; *LRL*, lower rhombic lip; *MG*, medial granule cell mass; *ML*, molecular layer; *MON*, medial nucleus; *Ob*, obex; *Sll*, sulcus longitudinalis lateralis; *URL*, upper rhombic lip

**Fig. 4:**
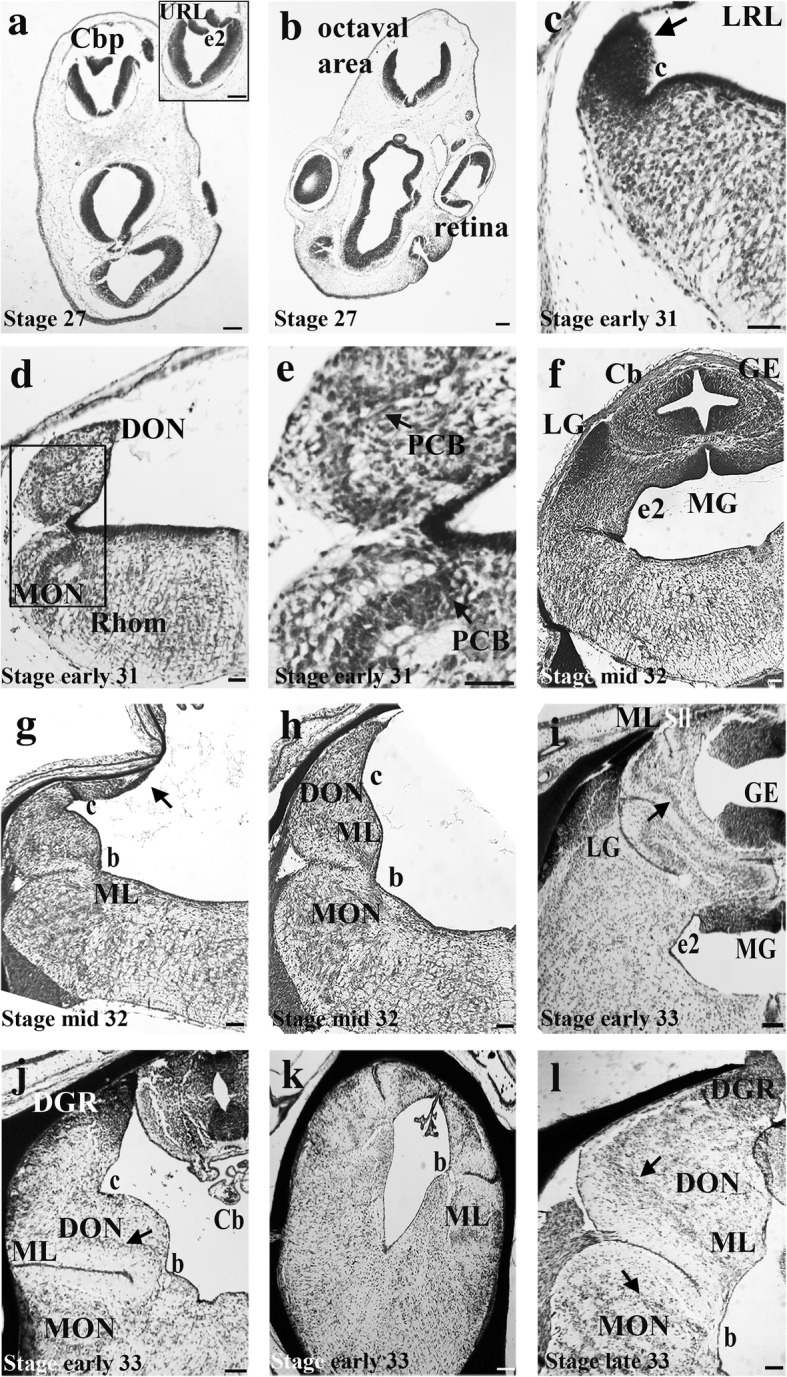
Light micrographs of *Leucoraja erinacea* sections showing cerebellar and cerebellar-like development. Stage 27 embryonic brain showing the incipient Cbp (**a**) and octaval area (**b**). Inset is a section between a and b, showing the URL and e2 sulcus. Early stage 31 brain showing the LRL and the incipient LG (**c**, arrow) with PCB’s in the DON (AENs) and MON (cell plate X) (**d**). Principal cell bands can be seen in inset from d, showing a higher magnification image of the PCB’s (**e**). Mid-stage 32 brain showing the incipient LG, MG, GE and cerebellar body (**f**), the DGR-like “sliver” of cells (**g**, arrow) and the newly formed DON and MON ML’s (**h**). Early stage 33 brain showing the cerebellar ML as well as LG, MG, GE and the Sll. Arrow points to Purkinje cell layer (**i**). Early stage 33 brain showing the newly formed DGR. Arrow points to PCB (**j**). Caudal section of the early stage 33 brain showing the ML extending past the obex (**k**). Late stage 33 brain showing the fully formed DGR, DON and MON MLs (arrows point to PCB) (**l**). Scale bars equal 100 μm. Abbreviations: *b*, sulcus b; *c*, sulcus c; *Cb*, cerebellum; *Cbp*, cerebellar plate; *DON*, dorsal nucleus; e2, sulcus e2; *GE*, granular eminences; *LG*, lateral granule cell mass; *LRL*, lower rhombic lip; *MG*, medial granule cell mass; *ML*, molecular layer; *MON*, medial nucleus; *PCB*, principal cell band; *Rhom*, rhombencephalon; *Sll*, sulcus longitudinalis lateralis; *URL*, upper rhomic lip

Neurologically, the cerebellar plate, which gives rise to the cerebellar body, first appears at this stage (Figs. 3, 4a). Caudal to the incipient cerebellar plate, the upper rhombic lip (URL) is present dorsally to a prominent sulcus, e2 (Figs. 3, 4a) [10]. The rhombencephalon shows little significant morphological variation along the rostral-caudal axis from the rostral end of the octaval area to the obex (Fig. 3, 4b).

### Early stage 31

At this stage in embryological development, the most noticeable trait is the gill filaments, which are at nearly their longest length relative to other developmental stages. The eyes are pigmented and start to shift dorsally. The mouth is rectangular with slight lateral upward curls. The pectoral fins continue to grow, but have not grown anterior to the eyes or fused with the gill regions. The pelvic fin develops its “butterfly” morphology. The dorsal fins are well developed. The angle between the forebrain and rostrum is noticeably smaller than at stage 30 (Fig. 2b) [12].

A dense cell mass is present caudal to the cerebellar plate and dorsal to sulcus e2, which corresponds to the incipient LG, as there is no dense cell mass caudal to this one which would represent DGR (Figs. 3, 4c, arrow). It is also possible that this cell mass instead subdivides into rostral and caudal components which will represent the future LG and DGR. Cell plate X, which is a dense band of projection cells in the mature MON, is first present at this stage and there is a band of cells within the DON which may correspond to the principal cell ascending efferent neuron (AEN) band (Figs. 3, 4d, e). It is possible that these are an unrelated transient mass of cells, but these cell bands are present in both the correct anatomical position and throughout subsequent embryological stages, indicating they may be principal cell bands.

### Mid-stage 32

The most identifiable characteristic of this stage is the fusion of the pectoral fin to the gill regions. The skin also acquires pigmentation during this stage. The eyes are located dorsally and the iris is fully pigmented black. The gill filaments are smaller than previous stages, indicating a retraction and a transition from external to internal gills. The mouth is slit-like (Fig. 2c) [12].

**Fig. 5:**
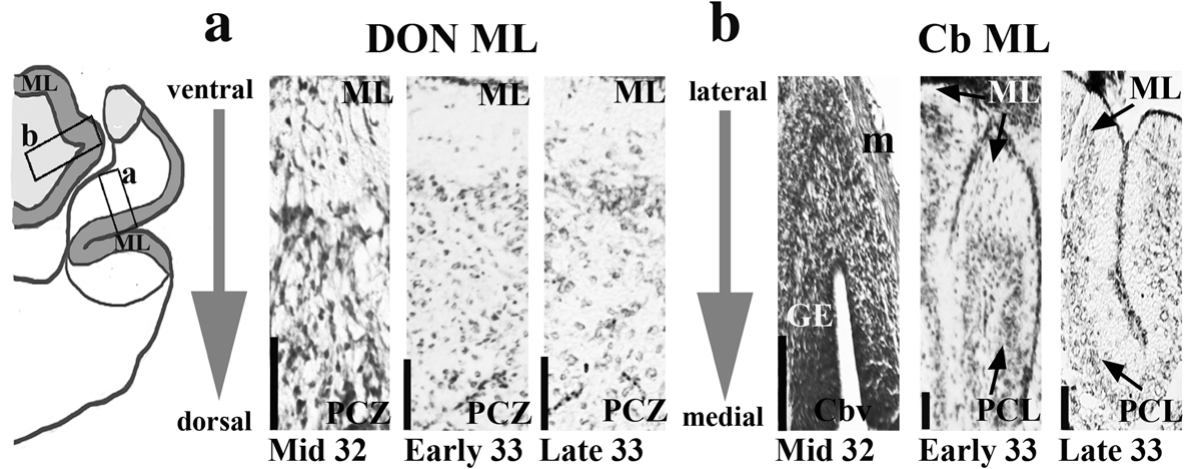
Light micrographs of cerebellar and cerebellar-like cross sections in *Leucoraja erinacea*, showing earlier ML development in the cerebellum-like structures than the Cb. Anatomical drawing illustrates the location of images in a and b. ML and PCZ in the DON at mid-stage 32, early stage 33 and late stage 33 (**a**). Sections are oriented ventral (ML) to dorsal (PCZ). Cb showing the ML and PCL at the same stages (**b**). Sections are oriented lateral (ML) to medial (GE). Scale bars equal 100 μm. Abbreviations: *Cb*, cerebellum; *Cbv*, cerebellar ventricle; *DON*, dorsal octavolateralis nucleus; *GE*, granular eminences; m, meninges; *ML*, molecular layer; *PCL*, Purkinje cell layer; *PCZ*, principal cell zone

At mid-stage 32, the LG, MG and GE are present but DGR has not formed (Fig. 3, 4f). There is a sliver-like mass of densely packed cells located caudally and dorsally near the choroid plexus in the octaval area, which may correspond to the first accumulation of granule cells in the DGR (Figs. 3, 4g). The morphology of the incipient MON and DON molecular layers are present at this stage, lateral to a prominent sulcus, b [10] (Figs. 3, 4h, 5a). Despite the development of the cerebellar body, including the GE, the morphology of the cerebellar molecular layer has not yet developed (Figs. 3, 4f, 5b). Figure 5 shows comparative cerebellar and cerebellar-like molecular layer development in stage 32 and 33 embryos. In these images, the molecular layer is located next to the principal cell zone of the DON and the Purkinje cell layer of the cerebellum. We define the development of the molecular layers by the presence of an area that has low cell density with interspersed small cells corresponding to interneurons in the correct anatomical position. It is possible that some cellular elements of the cerebellar molecular layer have already developed, but are not detected by our imaging technique.

### Early stage 33 (yolk present)

At early stage 33, the animal resembles a small juvenile, but the yolk has not been fully consumed. See inset from Fig. 2d for image of yolk. Pigmentation resembles that of an adult, although slightly lighter (Fig. 2d) [12].

The LG and MG appear larger and more differentiated at this stage (Figs. 3, 4i). Caudally, the LG grows dorsally, then splits into dorsal and ventral components. Both the dorsal and ventral components taper off, except for a small mass seen in the most dorso-medial region of the medulla. Caudally, this dorsal mass grows slightly and forms the incipient DGR (Figs. 3, 4j). The molecular layer of the MON and DON have not fully formed and are still only present lateral to sulcus b, although both are larger than at stage 32 (Figs. 3, 4j). The molecular layer extends caudally past the obex, though it does not continue to extend this far in the adult (Figs. 3, 4k). This may be the result of the molecular layer occupying a greater proportion of neural space in the embryo which diminishes as the rhombencephalon develops around it. The cerebellar molecular layer has formed, as has the Purkinje cell layer and the sulcus longitudinalis lateralis of the cerebellum (Figs. 3, 4i, 5b). At this stage, the Purkinje cells are densely packed and the Purkinje cell layer is several cells thick (Fig. 5b). The GE of the cerebellum has grown laterally. Rostrally, the cerebellar ventricle is large and diminishes caudally until that space is occupied by the medial granular eminences (Figs. 3, 4i).

### Late stage 33

At late stage 33, the yolk sac has been fully consumed and the animal resembles a small juvenile (Fig. 2e). The cerebellar molecular layer has fully developed. The DGR has fully formed and appears mature-like based on adult morphology (Figs. 3, 4l). Accordingly, the molecular layers of the MON and DON have also fully developed. The cerebellum will undergo some post-natal growth towards the obex, but the morphology is largely similar to that in newly hatched and adult skates.

## Discussion

### Cerebellar and cerebellar-like development in the little skate, *L. erinacea*

Here, we describe major developmental events in the dorsal hindbrain of the little skate. At stage 27, the cerebellar plate first develops. There is little morphological variation throughout the octaval area. At early stage 31, a dense cell mass develops representing the incipient LG. Principal cell bands first appear in the cerebellum-like structures. These principal cells are the functional analog of Purkinje cells in the cerebellum. The cerebellar-like principal cell bands are thus established before the development of their respective molecular layers, where they will extend their spiny apical dendrites. At mid-stage 32, the MG and GE of the cerebellum are present. The molecular layers of the cerebellum-like structures also develop at this stage. At early stage 33, the DGR has developed mature-like morphology and the cerebellar Purkinje cell and molecular layers develop. Thus, the LG develops before the DGR and the cerebellar-like molecular layers develop before the cerebellar molecular layer. A timeline describing major events of cerebellar and cerebellar-like development in the little skate are shown in Table 1.

**Table 1:** A timeline of comparative cerebellar and cerebellar-like development in *L. erinacea* and *S. canicula*

	St. 31	St. 32	St. 33
*L. erinacea*	LG	GE; MG; CbLS ML	Cb ML; DGR
*S. canicula*	UAL; LAL; GE; Cb ML	inter. Zone; CbLS ML	

Abbreviations: Cb ML, cerebellar molecular layer; CbLS ML, cerebellar-like molecular layer; DGR, dorsal granular ridge; GE, granular eminences of the cerebellum; inter. Zone, intermediate zone; LAL, lower auricular leaf; LG, lateral granule cell mass; MG, medial granule cell mass; UAL, upper auricular leaf

### Comparisons to dorsal hindbrain development in the shark, *Scyliorhinus canicula*

Hindbrain development has been studied in the spotted catshark, *Scyliorhinus canicula*, by Pose-Mendez et al. 2016 [5, 13]. However, conducting a direct comparison between shark and skate hindbrain development presents some difficulties. Pose-Mendez et al. 2016 [5] and Rodriguez-Moldes et al. 2008 [13] define the regions of the dorsal rhombencephalon by the borders of restricted gene expression and not functional anatomical connections, so there may be some discrepancy in the strict definitions of the relative structures between shark [5, 13] and skate [8] (Table 2). One example is the term ‘auricles,’ which is commonly used in the shark, instead of the MG or LG, which are used in the skate. MG corresponds to the medial region of the UAL and LG may be homologous to part of the LAL. The DGR may be homologous to a region termed the intermediate zone, which lies ventrally to the LAL and dorsally to the DON in the adult shark. This is confirmed with *HoxA2* and *En2* expression data by Pose-Mendez et al. 2016 [5]. Another difference is the term cerebellar crest, used to identify the molecular layer of the cerebellum-like structures in the shark, while the term molecular layer is used in both the cerebellum and cerebellum-like structures of the skate.

**Table 2:** Terminology of corresponding granule cell masses between *L*. *erinacea* and *S*. *canicula*

*L*. *erinacea*	*S*. *canicula*
Medial granule cell mass (MG)	Upper auricular leaf (UAL)
Lateral granule cell mass (LG)	Lower auricular leaf (LAL)
Dorsal granular ridge (DGR)	Intermediate zone
Granular eminences of the cerebellum (GE)	Granular eminences of the cerebellum (GE)

In addition to differences in anatomical labeling, the exact definitions of the developmental stages may not correspond exactly between the species as they are based on external morphology, which differs between shark and skate. Despite these differences in external morphology, developmental patterns and sequences for the cerebellum and cerebellum-like structures are largely similar between *S. canicula* and *L. erinacea* (Table 1).

Briefly, cerebellar development in both species can be split into three periods. During the first phase, the cerebellar plate develops from the URL. During the second phase, the cerebellar body develops. During the third phase, the cerebellum and related areas develop mature-like morphology (Table 1) [5].

There are also shared aspects of cerebellar-like development between the shark and skate. In the skate, the LG develops before the DGR. The corresponding regions develop in the same manner (UAL and intermediate zone, respectively) in the shark. The cerebellar-like molecular layers in both species also develop at stage 32 (Table 1).

Dorsal hindbrain anatomy in the adult stage is also comparable between *L. erinacea*, *S. canicula*, and that of *Squalus acanthias* as described by Larsell [14]. In the adult stage, the morphology of LG appears similar among these species. The morphology of the DGR and the GE of the cerebellum is more similar between the sharks, *S. canicula* and *S. acanthias*, relative to the skate *L. erinacea*. A major cerebellar sulcus, the sulcus longitudinalis lateralis, appears to be present in *L. erinacea*, but not *S. canicula* or *S. acanthias* and is a differentiating feature of batoids from sharks [10]. Despite these gross morphological differences, the micro-circuitry that comprises these cerebella and cerebellum-like structures is very well conserved [5, 15, 16].

### Genesis of the DGR and DON by duplication

**Fig. 6:**
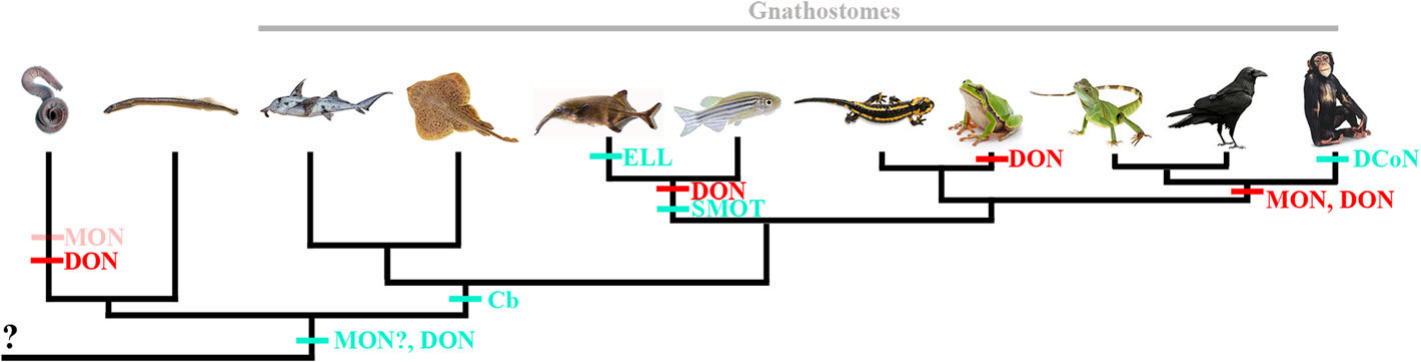
Vertebrate phylogeny showing the presence of the cerebellum and cerebellum-like structures. Presence of structures is marked by blue, and loss is marked by red. Light red in the hagfish lineage represents a partial loss of the MON. From left to right, animals are representatives from Agnatha, Holocephali and Elasmobranchii (Chondrichthyes), Mormyridae and Cyprinidae (Actinopterygii), Urodela and Anura (Amphibia), Reptilia, Aves, and Mammalia. *Cb*, cerebellum; *DCoN*, dorsal cochlear nucleus; *DON*, dorsal nucleus; *ELL*, electrosensory lateral lobe; *MON*, medial nucleus; *SMOT*, stratum marginale of the optic tectum

It has previously been shown that the cerebellum, DON, and MON of the little skate utilize features of a shared developmental toolkit, visualized by common gene expression and protein localization. Granule cells in cerebellar and cerebellar-like structures co-express *Pax*6 and *Cbln1*, while principal cells in cerebellar and cerebellar-like structures express *Grid*2 [6]. These results, together with the phylogenetic presence of these structures, indicates that the cerebellum may have evolved as a result of the duplication or expansion of the cerebellar-like developmental genetic toolkit [3, 6] (Fig. 6). Although the cerebellum may have evolved due to duplication of the DON and MON developmental toolkit, it remains unknown whether the DON or MON is the ancestral cerebellar-like nucleus. Developmental and morphological data presented here and in other reports [5, 6, 11] may provide insight into the evolutionary genesis of the cerebellar-like structures.

The DGR develops after the LG in both shark and skate (Table 1). This suggests that the evolutionary genesis of the DGR may then be the result of a caudal and temporal expansion of the developmental program responsible for the development of the LG. To examine this possibility, we must consider the connections of these granule cell masses to the first order sensory nuclei and their sensory organs. The DGR, DON, and electroreceptors are an integral part of the electrosensory system while the LG, MON, and mechanosensory hair cells are part of the lateral line system. Both the mechanosensory lateral line system and electrosensory system are present in chondrichthyans and some members of the gnathostome out group; lampreys possess an electrosense and a mechanosensory lateral line with a DON and MON, which appear to be cerebellar-like (Fig. 6) [17]. Eptatretid hagfish have a simplified lateral line system, myxinoid hagfish do not have a lateral line system and neither hagfish possess an electrosense (Fig. 6) [18, 19]. Thus, the electrosense may be an evolutionarily newer sensory system, or has been lost within the hagfish lineage.

It is known that mechanosensory hair cells are present in the coronal organ of ascidians and the epidermis of amphioxus [20, 21]. Similar to the hair cells of the vertebrate lateral line and electrosense, these hair cells are derived from neurogenic placodes and may be homologous to vertebrate mechanosensory hair cells, implying that mechanosensing is representative of the ancestral function for these hair cells. If true, the neural regions that are responsible for processing mechanosensory information would be ancestral to regions that process electrosensory information.

Although speculative, we hypothesize that the MON may be the ancestral nucleus, from which other cerebellum-like structures emerged (Fig. 6). It is also possible that the delay in elasmobranch DGR development may simply be the result of dorsal rhombencephalic development occurring in a rostral-caudal fashion from the mid-hindbrain boundary, rather than an indicator of evolutionary history. It is also possible that excitatory granule cells of the DGR are generated in the more rostral URL with subsequent caudal migration to form the DGR. The rhombic lip does appear to be present in eptatrid hagfish and an examination of cerebellar-like development in agnathans will shed insight into the genesis of cerebellar/cerebellar-like evolution [22].

## Conclusions

The development of the dorsal hindbrain in *L. erinacea* is described. The development of the LG, DGR, MG, GE of the cerebellum and their respective molecular layers is of particular importance for our understanding of comparative cerebellar development. The LG, MG, and cerebellar body fully develop in stage 32 with the DGR following in early stage 33. The cerebellar-like molecular layers develop in stage 32 with the cerebellar molecular layer following in stage 33. This pattern is similar to hindbrain development in *S. canicula*, and together with molecular toolkit data, contributes to our understanding of how the MON, DON and cerebellum may have evolved through duplication of a common developmental program.

## Abbreviations

b: Sulcus b
c: Sulcus c
Cb: Cerebellum
Cbp: Cerebellar plate
DGR: Dorsal granular ridge
DON: Dorsal nucleus
e2: Sulcus e2
GE: Granular eminences
LG: Lateral granule mass
LRL: Lower rhombic lip
MG: Medial granule cell mass
ML: Molecular layer
MON: Medial nucleus
Ob: Obex
Sll: Sulcus longitudinalis lateralis
URL: Upper rhombic lip
